# *AAVS1* ‐targeted, stable expression of ChR2 in human brain organoids for consistent optogenetic control

**DOI:** 10.1002/btm2.10690

**Published:** 2024-06-09

**Authors:** Soojung Hong, Juhee Lee, Yunhee Kim, Eunjee Kim, Kunyoo Shin

**Affiliations:** ^1^ School of Biological Sciences, College of Natural Sciences, Seoul National University Seoul Republic of Korea; ^2^ Institute of Molecular Biology and Genetics, Seoul National University Seoul Republic of Korea

**Keywords:** *AAVS1* locus, channelrhodopsin‐2, forebrain organoids, optogenetics

## Abstract

Self‐organizing brain organoids provide a promising tool for studying human development and disease. Here we created human forebrain organoids with stable and homogeneous expression of channelrhodopsin‐2 (ChR2) by generating *AAVS1* safe harbor locus‐targeted, ChR2 knocked‐in human pluripotent stem cells (hPSCs), followed by the differentiation of these genetically engineered hPSCs into forebrain organoids. The resulting ChR2‐expressing human forebrain organoids showed homogeneous cellular expression of ChR2 throughout entire regions without any structural and functional perturbations and displayed consistent and robust neural activation upon light stimulation, allowing for the non‐virus mediated, spatiotemporal optogenetic control of neural activities. Furthermore, in the hybrid platform in which brain organoids are connected with spinal cord organoids and skeletal muscle spheroids, ChR2 knocked‐in forebrain organoids induced strong and consistent muscle contraction upon brain‐specific optogenetic stimulation. Our study thus provides a novel, non‐virus mediated, preclinical human organoid system for light‐inducible, consistent control of neural activities to study neural circuits and dynamics in normal and disease‐specific human brains as well as neural connections between brain and other peripheral tissues.

**Translational Impact Statement**

We created human forebrain organoids that stably express ChR2 throughout entire regions of the organoids during long‐term culture, which showed a non‐virus mediated, robust neural activation upon light stimulation and further induced strong and consistent muscle contraction in the hybrid assembloid platform, being connected to spinal cord organoids and skeletal muscles spheroids. Our ChR2‐engineered forebrain organoids will serve as an innovative experimental platform to study neural circuits in normal and patient‐specific brains, and further provide a unique optogenetic tool for the development of new therapeutic options that may be customized for individual patients with various neurological disorders.

## 1 INTRODUCTION

Understanding human brain development and neurological disease is challenging due to the scarcity and inaccessibility of human brain tissues. With increasing advance of stem cell technology and three‐dimensional (3D) cultures, hPSCs, including human embryonic stem cells and human induced pluripotent stem cells, can be developed into self‐organizing 3D tissues in vitro representing the human brain, called brain organoids.^1^, ^2^, ^3^, ^4^ Not only whole‐brain organoids,^2^, ^3^, ^5^ but also region‐specific organoids, such as forebrain organoids,^4^, ^6^, ^7^ cerebellum organoids,^8^ midbrain organoids,^9^ thalamus organoids,^10^ and hypothalamus organoids,^6^ have been developed for several years. In contrast to conventional two‐dimensional (2D) cell cultures and genetically‐engineered mouse models, which do not precisely mimic the complex and dynamic cellular interactions of the human brain, brain organoids represent several aspects of the human brain in vivo, such as tissue architecture and cell composition, thereby serving as a valuable model for studying neural circuits and dynamics in the human brain as well as in the context of brain disease.^2^, ^11^, ^12^, ^13^, ^14^, ^15^, ^16^ Furthermore, combining brain organoids with other region‐specific organoids or various tissue organoids to generate hybrid assembloids provides a powerful platform for studying neural migration and networks across different brain regions and modeling anatomical and functional connectivity between distinct tissues.^10^, ^17^, ^18^, ^19^, ^20^, ^21^

Optogenetics is a technique to control the activity of genetically‐engineered cell populations by introducing light‐sensitive proteins, such as ion channels, pumps, or enzymes, which enables spatiotemporal activation or inhibition of cells of interest using light.^22^, ^23^, ^24^ Optogenetic approaches have been successfully applied in vitro to study neural networks and synaptic functions within certain neural populations^20^, ^25^, ^26^, ^27^, ^28^, ^29^, ^30^ as well as in vivo to understand neural circuits and the role of specific brain regions in memory and behavior,^23^, ^31^, ^32^ revolutionizing the neuroscience field over the past decade.

To introduce transgenes encoding light‐sensitive proteins into cells of interest, viral delivery is commonly used in optogenetic experiments.^7^, ^25^, ^26^, ^28^, ^29^, ^32^, ^33^, ^34^, ^35^, ^36^ Although viral infection provides a convenient way to deliver transgenes into cells, it often leads to inconsistent and heterogeneous gene expressions that can be easily silenced. Also, random integration of lentiviral delivery systems may alter endogenous gene expressions, which in turn can affect both normal and disease phenotypes.^37^, ^38^ It is possible to overcome this limitation using adeno‐associated virus (AAV), but transient gene expressions by AAV delivery limit further experiments that require long‐term culture.^37^ As such, current viral delivery systems do not allow precise and robust control of neural activities.

Targeted integration of transgenes into defined loci hinders random interference with other gene elements, allowing safe and homogeneous transgene expressions. In combination with the CRISPR/Cas9 system, “donor” plasmids with transgenes flanked by genomic homology regions are employed to mediate cellular homology‐directed repair (HDR) pathways.^39^ CRISPR/Cas9 induce double‐strand breaks at target sites in the genome, activating cellular intrinsic DNA damage response and repair pathways.^40^, ^41^ In contrast to non‐homology end‐joining (NHEJ), in which broken ends of DNA are joined together to generate insertions and deletions (known as ‘indels’),^42^, ^43^ donor‐mediated HDR pathways faithfully reconstruct the genomic sequence, allowing precise integration of genes of interest in a locus‐specific manner.

The adeno‐associated virus integration site 1 (*AAVS1*) locus, which is located in intron 1 of *PPP1R12C* (protein phosphatase 1 regulatory subunit 12C) on human chromosome 19, was originally described as a main hotspot for AAV integration. Because of its transcription‐competent, open chromatin structure without any known evidence to suggest adverse effects associated with its disruption,^44^, ^45^ *AAVS1* is considered a “safe harbor” for exogenously inserted gene expression. Thus, targeted integration of transgenes into *AAVS1* locus via homologous recombination with CRISPR/Cas9 enables stable and long‐term expression of transgenes in various cell types, including hPSCs.^44^, ^45^, ^46^, ^47^

In this study, we generated an efficient optogenetic model of forebrain organoids with homogeneous and stable expression of ChR2 by selectively introducing ChR2 into the *AAVS1* locus of hPSCs. These ChR2‐engineered forebrain organoids showed robust neural activation upon light stimulation and further induced strong and consistent muscle contraction when fused with spinal cord organoids and skeletal muscle spheroids to generate cortico‐spinal‐muscle assembloids. Therefore, our ChR2‐engineered forebrain organoids provide a valuable platform for optogenetic experiments, enhancing our understanding of neural circuits and dynamics in the human brain and within various neurological diseases.

## 2 RESULTS

### 2.1 Generation of *AAVS1* safe harbor locus‐targeted, ChR2 knocked‐in hPSC lines

**FIGURE 1:**
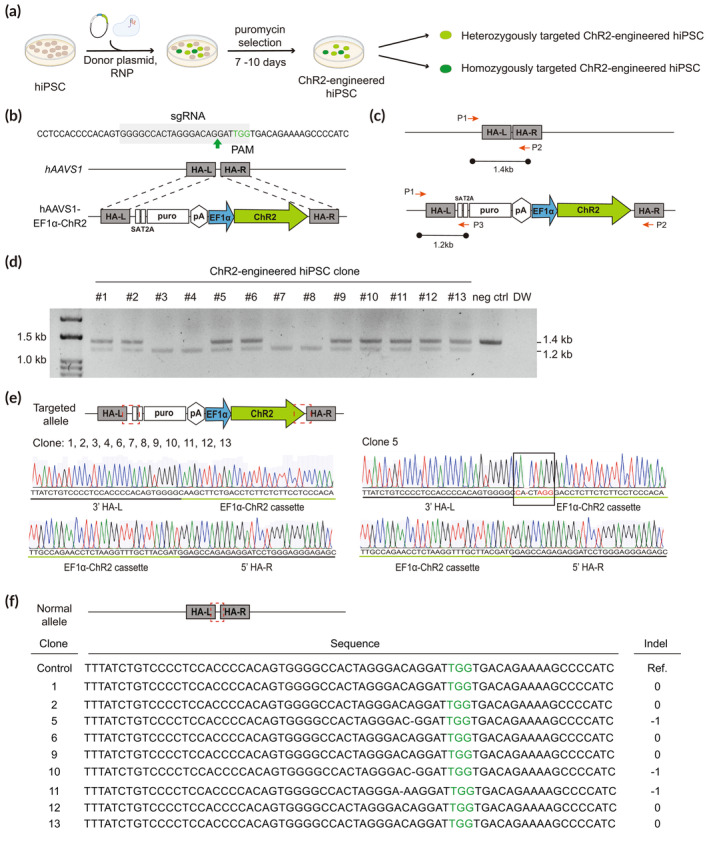
Establishment of ChR2‐engineered hPSC lines by targeted introduction of EF1α‐ChR2 into the *AAVS1* locus. (a) Experimental scheme to generate ChR2‐engineered hPSCs using the donor plasmid and RNP system. Heterozygously targeted and homozygously targeted hPSC clones are obtained after puromycin selection. (b) Schematic illustration of the *AAVS1* locus targeted with EF1α‐ChR2. (c) Genotyping strategies to identify targeted hPSC clones. PCR primers were designed to detect normal and targeted *AAVS1* alleles, whose amplification results in 1.4 and 1.2 kbp fragments, respectively. (d) PCR genotyping results of ChR2‐engineered hPSC clones by the amplification of 3 primers: P1, P2, and P3. The 1.4 kbp band indicates the presence of normal alleles, whereas the 1.2 kbp band represents the targeted allele. (e) The sequencing results of the recombination sites of ChR2‐engineered hPSC clones. (f) The sequencing results of the recombination sites at the non‐targeted allele of ChR2‐engineered, heterozygous clones.

Optogenetic approaches using conventional viral systems to deliver transgenes encoding light‐sensitive proteins have several limitations, such as inducing inconsistent and heterogeneous gene expressions that can be readily silenced per experiment and altering endogenous gene expression due to random integration.^37^, ^38^ In this study, we sought to establish hPSC lines in which the ChR2 gene is stably knocked in the *AAVS1* safe harbor locus to elicit stable and homogeneous expression of ChR2 (Figure 1a). To achieve this, a donor plasmid harboring the ChR2 gene with the EF1α promoter was designed, allowing robust gene expression under the control of the EF1α promoter in pluripotent stem cells as well as differentiated cells (Figure 1b). Donor plasmid was then introduced into hPSCs together with a ribonucleoprotein (RNP) complex consisting of the Cas9 protein and a single guide RNA (sgRNA) targeting *AAVS1* by electroporation (Figure 1a,b). After 2 weeks of puromycin selection, a total of 13 hPSC colonies survived, and their genotypes were determined by PCR (Table S1). To confirm the targeted integration of the transgenes into the *AAVS1* locus, PCR primers were designed to detect normal and targeted *AAVS1* alleles, whose amplification results in 1.4 and 1.2 kbp fragments, respectively (Figure 1c). PCR genotyping analysis showed that four clones were homozygous, where ChR2 was introduced into *AAVS1* of both alleles, and nine clones were heterozygous, carrying one copy of targeted allele and one normal allele (Figure 1d).

To verify whether faithful integration of EF1α‐ChR2 cassette without any mutations had occurred, we amplified and sequenced the region near the recombination site in the targeted allele (Figure 1e). We found that only clone 5 had a few mismatches in front of the EF1α‐ChR2 cassette, leading to a frameshift of ChR2 (Figure 1e). To further rule out the possibility of heterozygous clones acquiring any mutations from NHEJ in normal alleles, the region where recombination occurs—in between the left and right homology arms of the *AAVS1* locus—was amplified and sequenced (Figure 1f). We found that clones 5, 10, and 11 had deletions near the PAM site, but other clones did not show any mutations (Figure 1f). Taken together, we successfully generated six heterozygous and four homozygous clones where the EF1α‐ChR2 cassette was faithfully introduced into the *AAVS1* locus without any mutations.

### 2.2 Homogeneous and stable expression of functional ChR2 proteins in ChR2‐engineered hPSCs

**FIGURE 2:**
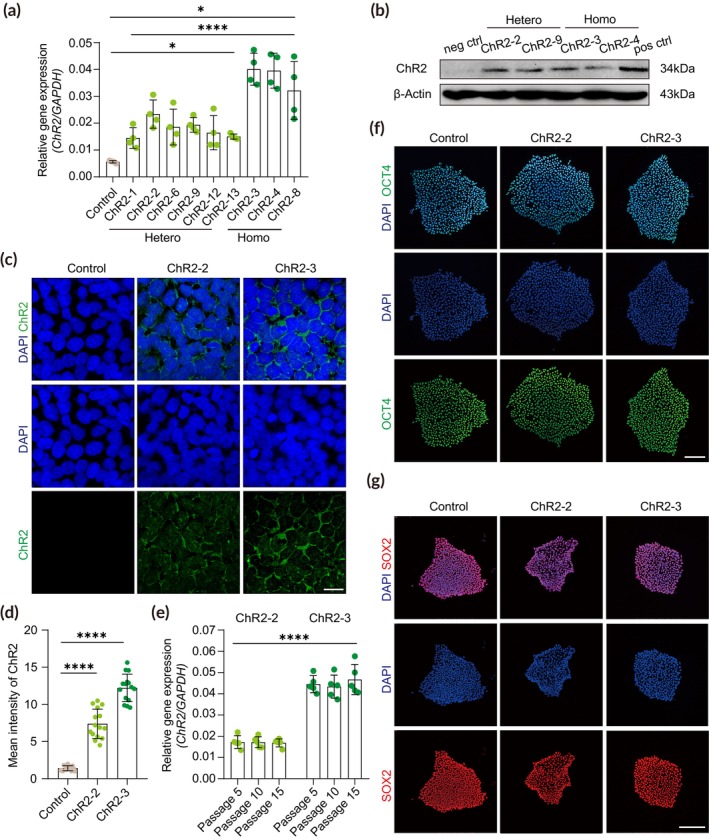
Homogeneous and stable expression of ChR2 in ChR2‐engineered hPSCs. (a) Relative expressions of ChR2 in ChR2‐engineered and control hPSCs. ChR2‐1, 2, 6, 9, 12, and 13, heterozygous clones; ChR2‐3, 4, 7, and 8, homozygous clones. Four technical replicates were evaluated for each line (*n* = 4). Significance was calculated using two‐tailed nested *t*‐test. (b) Western blot analyses of ChR2‐engineered hPSCs. ChR2‐2 and 9, heterozygous clones; ChR2‐3 and 4, homozygous clones; neg ctrl, negative control (non‐targeted hPSC); pos ctrl, positive control (HEK293 cells transfected with ChR2). (c) Immunofluorescence analysis for ChR2 in control hPSCs and ChR2‐engineered hPSC lines (ChR2‐2, ChR2‐3). Scale bar, 20 μm. (d) Quantification of relative ChR2 expression level in control hPSCs and ChR2‐engineered hPSC lines (ChR2‐2, ChR2‐3). Each dot represents the mean intensity of the ROI. Three ROIs from five colonies were analyzed (*n* = 15). Significance was calculated using an unpaired *t‐*test. (e) Relative expressions of ChR2 in ChR2‐engineered hPSC lines (ChR2‐2, ChR2‐3) following passages. Five technical replicates were evaluated in each group (*n* = 5). Significance was calculated using two‐tailed nested *t*‐test. (f) Immunofluorescence analysis of the pluripotency marker OCT4 in control hPSCs and ChR2‐engineered hPSC lines (ChR2‐2, ChR2‐3). Scale bar, 100 μm. (g) Immunofluorescence analysis of the pluripotency marker SOX2 in control hPSCs and ChR2‐engineered hPSC lines (ChR2‐2, ChR2‐3). Scale bar, 100 μm.

To confirm the expression of ChR2 at the gene level, we performed RT‐qPCR of ChR2‐engineered, 6 heterozygous (ChR2‐1, 2, 6, 9, 12, 13) and 4 homozygous (ChR2‐3, 4, 7, 8) hPSC clones (Table S1). All of the ChR2‐engineered clones showed higher expression of ChR2 than the non‐targeted control (Figure 2a). Of note, homozygous clones showed gene expressions that were two times greater than those of heterozygous clones. To examine the expression of ChR2 at the protein level, we performed western blot analysis using the ChR2 antibody. We found robust expression of functional ChR2 proteins in both heterozygous and homozygous clones, displaying distinct bands at ~34 kDa corresponding to the full‐length monomer protein (Figure 2b).^48^

We then examined the expression patterns and the cellular localization of ChR2 proteins by immunocytochemistry. In contrast to the non‐targeted control, ChR2 was homogeneously expressed in ChR2‐engineered hPSCs; the mean intensity of the homozygous clone ChR2‐3 was approximately two times greater than that of the heterozygous clone ChR2‐2 (Figure 2c,d). Also, the localization of ChR2 was restricted to the cell membrane (Figure 2c). To test whether ChR2 expression is maintained for an extended time, the ChR2 expression levels in ChR2‐2 and ChR2‐3 clones were examined across multiple passages. We found stable and consistent expressions of ChR2 throughout passages 5, 10, and 15 in both clones (Figure 2e), which also maintained pluripotency, expressing pluripotency markers OCT4 and SOX2 (Figure 2f,g). Overall, we established a robust system to generate hPSC lines that homogeneously express ChR2 with pluripotency during long‐term culture, which can be further utilized to differentiate into various types of cells or tissues.

### 2.3 ChR2‐expressing forebrain organoids represent NPC proliferation and early neurogenesis

**FIGURE 3:**
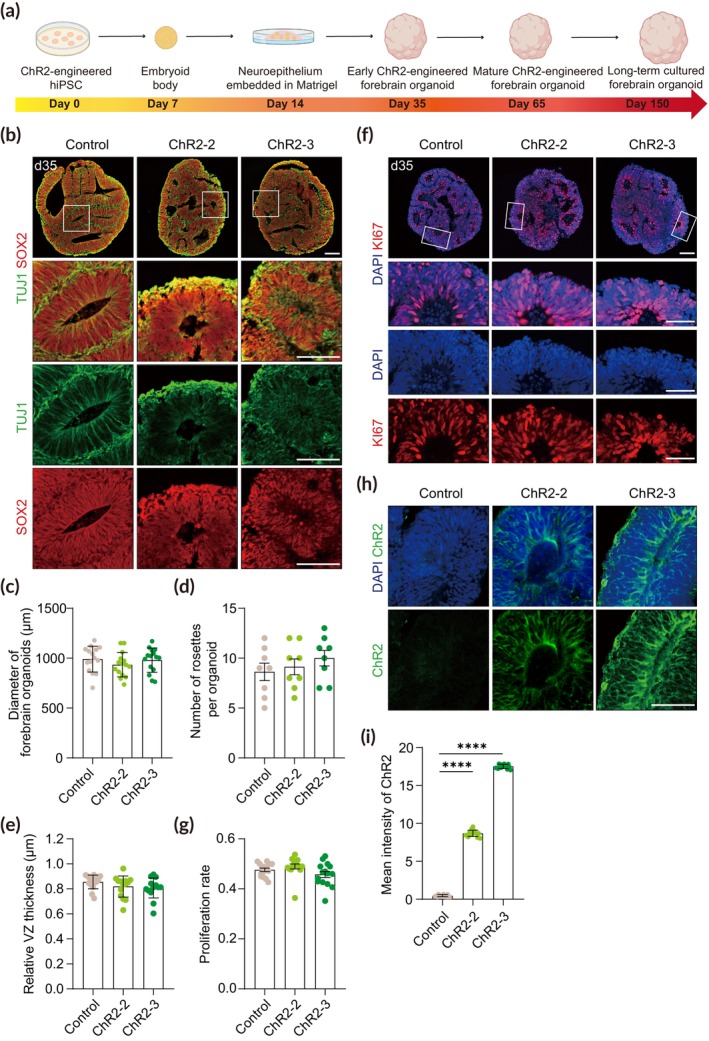
Characterization of ChR2‐engineered forebrain organoids at the early stage. (a) Experimental scheme to generate ChR2‐engineered forebrain organoids. (b) Representative images of forebrain organoids at day 35 immunostained for the NPC marker SOX2 and the neuronal marker TUJ1. Scale bar, 100 μm. (c) Quantification of the diameter of control and ChR2‐engineered forebrain organoids. Forebrain organoids were imaged with brightfield and evaluated (*n* = 15). Significance was calculated using an unpaired *t*‐test. (d) Quantification of the number of rosettes in control and ChR2‐engineered forebrain organoids. Two sections per sample, four biological replicates in each group were evaluated (*n* = 8). Significance was calculated using an unpaired *t*‐test. (e) Quantification of the relative VZ thickness of control and ChR2‐engineered forebrain organoids. Five rosettes per sample, three biological replicates were analyzed (*n* = 15). Significance was calculated using an unpaired *t*‐test. (f) Representative images of forebrain organoids at day 35 immunostained for the proliferation marker Ki67. Scale bar, 100 μm. (g) Quantification of the proliferation rate using Ki67^+^ cells of DAPI^+^ cells. Five rosettes per sample, three biological replicates in each group were evaluated (*n* = 15). Significance was calculated using an unpaired *t*‐test. (h) Representative images of forebrain organoids at day 35 immunostained for ChR2. Scale bar, 50 μm. (i) Quantification of ChR2 intensity in control, ChR2‐2, and ChR2‐3 forebrain organoids. Five sections per sample, three biological replicates in each group were evaluated (*n* = 15). Significance was calculated using an unpaired *t*‐test.

We next generated forebrain organoids from ChR2‐engineered hPSCs—one of each from the heterozygous (ChR2‐2) and homozygous (ChR2‐3) lines (Figure 3a). Immunofluorescence analysis of forebrain organoids at the early stage showed well‐defined, ventricular zone (VZ)‐like structures of multiple rosettes packed with SOX2^+^ neural progenitor cells (NPCs) and outer neuronal layers consisting of TUJ1^+^ premature neurons, indicating early neurogenesis (Figure 3b). We found no significant differences in the size of forebrain organoids, the number of rosettes per organoids, and the relative thickness of the VZ in ChR2‐engineered forebrain organoids compared to the non‐targeted control (Figure 3c–e). In addition, immunofluorescence analysis with the Ki67 marker showed that proliferation of NPCs was similar between the control and ChR2‐engineered organoids (Figure 3f,g).

To confirm the expression of ChR2 in ChR2‐engineered forebrain organoids, we analyzed these organoids by immunofluorescence. We found homogeneous expression of ChR2 on the cell surface throughout entire cells only in ChR2‐engineered forebrain organoids but not in control organoids (Figure 3h). Forebrain organoids derived from the ChR2‐3 homozygous line showed ~2‐fold increase in ChR2 expression than forebrain organoids of the ChR2‐2 heterozygous line (Figure 3i).

### 2.4 ChR2‐engineered forebrain organoids exhibit homogeneous and stable expression of ChR2 throughout entire mature neurons over an extended culture period

**FIGURE 4:**
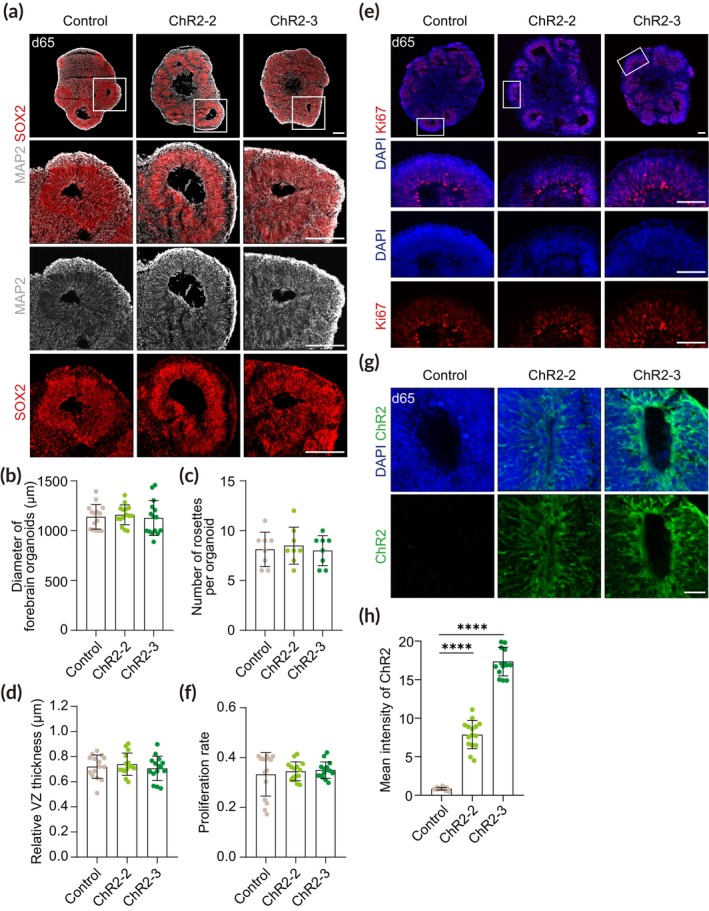
Characterization of ChR2‐engineered forebrain organoids at the late stage. (a) Representative images of forebrain organoids at day 65 immunostained for SOX2 and MAP2. Scale bar, 100 μm. (b) Quantification of the diameter of control and ChR2‐engineered forebrain organoids. Forebrain organoids were imaged with brightfield and evaluated (*n* = 15). Significance was calculated using an unpaired *t*‐test. (c) Quantification of the number of rosettes in control and ChR2‐engineered forebrain organoids. Two sections per sample, four biological replicates in each group were evaluated (*n* = 8). Significance was calculated using an unpaired *t*‐test. (d) Quantification of the relative VZ thickness of control and ChR2‐engineered forebrain organoids. Five rosettes per sample, three biological replicates were analyzed (*n* = 15). Significance was calculated using an unpaired *t*‐test. (e) Representative images of forebrain organoids at day 65 immunostained for Ki67. Scale bar, 100 μm. (f) Quantification of the proliferation rate using Ki67^+^ cells of DAPI^+^ cells. Five rosettes per sample, three biological replicates in each group were evaluated (*n* = 15). Significance was calculated using an unpaired *t*‐test. (g) Representative images of forebrain organoids at day 65 immunostained for ChR2. Scale bar, 50 μm. (h) Quantification of ChR2 intensity in control, ChR2‐2, and ChR2‐3 forebrain organoids. Five sections per sample, three biological replicates in each group were evaluated (*n* = 15). Significance was calculated using an unpaired *t‐*test.

To assess whether ChR2‐engineered forebrain organoids can further develop and undergo neuronal differentiation, we analyzed our ChR2‐engineered forebrain organoids by immunofluorescence at the late stage. Similar to control organoids, we observed the cortical plate‐like structures containing MAP2^+^ mature neurons formed above SOX2^+^ VZ structures in ChR2‐engineered forebrain organoids at day 65 (Figure 4a). The overall size of forebrain organoids was slightly increased, and the relative thickness of the VZ was reduced in all groups compared to early‐stage organoids due to the active proliferation of NPCs at the early stage of development (Figures 3b,d and 4b,d). However, in line with the previous data from early‐stage organoids, there were no significant differences in the size of forebrain organoids, the number of rosettes per organoid, and the relative VZ thickness between ChR2‐engineered and non‐targeted control organoids (Figure 4b–d). In addition, we observed no significant difference in NPC proliferation between the ChR2‐engineered and non‐targeted control organoids despite a slight decrease compared to early‐stage organoids (Figure 4e,f).

We further examined ChR2 expression in the late‐stage forebrain organoids at day 65. Similar to early‐stage organoids, ChR2 was localized at the cell surface and homogeneously expressed throughout the entire cells including mature neurons residing in the outer layer of the VZ in ChR2‐engineered forebrain organoids (Figure 4g). The expression of ChR2 was maintained at similar levels as early‐stage organoids and its fluorescence intensity in forebrain organoids from the homozygous ChR2‐3 line was nearly twice as high as that in forebrain organoids from the heterozygous ChR2‐2 line, consistent to the previous data from early‐stage organoids (Figures 3h,i and 4g,h).

**FIGURE 5:**
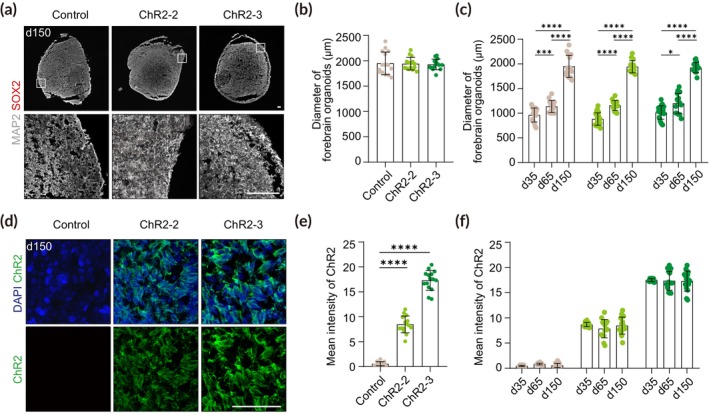
Comparative analysis of ChR2‐engineered forebrain organoids over an extended culture period. (a) Representative images of forebrain organoids at day 150 immunostained for SOX2 and MAP2. Scale bar, 100 μm. (b) Quantification of the diameter of control and ChR2‐engineered forebrain organoids. Forebrain organoids were imaged with brightfield and evaluated (*n* = 15). Significance was calculated using an unpaired *t‐*test. (c) Quantification of the diameter of control and ChR2‐engineered forebrain organoids on days 35, 65 and 150. Forebrain organoids were imaged with brightfield and evaluated (*n* = 15). Significance was calculated using an unpaired *t‐*test. (d) Representative images of forebrain organoids at day 150 immunostained for ChR2. Scale bar, 50 μm. (e) Quantification of ChR2 intensity in control and ChR2‐engineered forebrain organoids. Five sections per sample, three biological replicates in each group were evaluated (*n* = 15). Significance was calculated using an unpaired *t‐*test. (f) Quantification of ChR2 intensity in control and ChR2‐engineered forebrain organoids on days 35, 65 and 150. Five sections per sample, three biological replicates in each group were evaluated (*n* = 15). Significance was calculated using an unpaired *t‐*test.

To further explore whether ChR2 expression is maintained in ChR2‐engineered forebrain organoids for extended culture periods, we evaluated the ChR2 expression as well as the overall brain structure in 150‐day‐old ChR2‐engineered forebrain organoids. Despite the development of some cleaved caspase‐3^+^ necrotic areas (Figure S1a–d), the organoids continued to expand, with their size increasing up to day 150 (Figure 5a–c). These organoids predominantly consisted of MAP2^+^ mature neurons without visible SOX2^+^ VZ structures, consistent with previous findings^49^, ^50^ (Figure 5a). Moreover, ChR2 expression remained consistent and stable across the entire cortical area in these long‐term cultured, 150‐day‐old organoids (Figure 5d–f).

Taken together, our findings indicate that forebrain organoids differentiated from ChR2‐engineered hPSCs mimic the tissue architecture of the human brain in vivo including VZ‐like structures and the outer neuronal layer without any structural and functional perturbations, and show stable and homogeneous expression of ChR2 throughout the entire regions of the organoids during long‐term culture.

### 2.5 ChR2‐engineered forebrain organoids exhibit neural activation upon optogenetic stimulation

**FIGURE 6:**
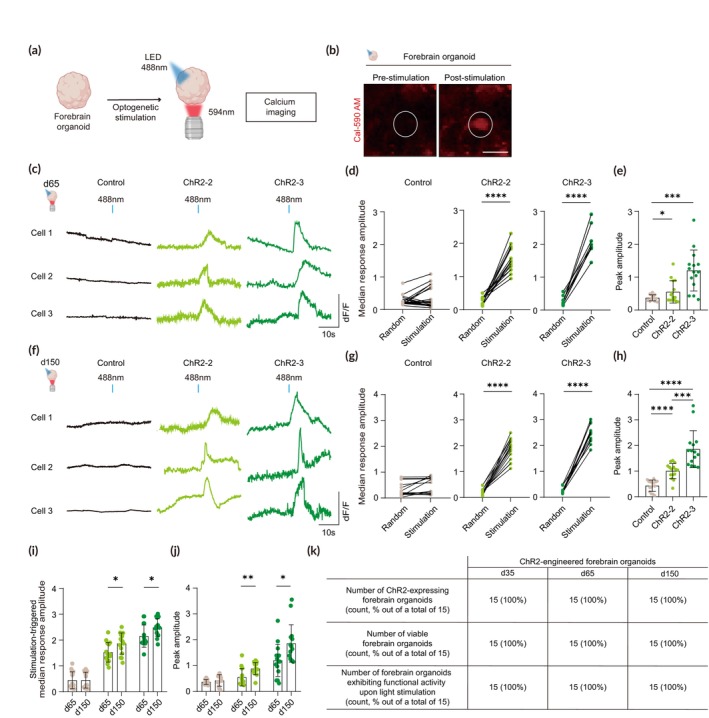
Optogenetic control of neural activities in ChR2‐engineered forebrain organoids. (a) Schematic illustration to examine neural activation upon optogenetic stimulation of ChR2‐engineered forebrain organoids by calcium imaging using Cal‐590 AM. (b) Representative images of Cal‐590 AM in ChR2‐engineered forebrain organoids before and after light stimulation. Scale bar, 5 μm. (c) Representative traces of calcium imaging analysis of selected cells following light stimulation of control and ChR2‐engineered forebrain organoids. (d) Quantification of stimulation‐triggered amplitudes, shown in comparison to randomized‐triggered amplitudes in control and ChR2‐engineered forebrain organoids. The median amplitude of the five pulses delivered per cell is shown. Five cells per sample, three to four biological replicates were evaluated in each group (Control, *n* = 20; ChR2‐2, *n* = 15; ChR2‐3, *n* = 15). Significance was calculated using an unpaired *t‐*test. (e) Quantification of peak amplitudes by measuring the relative changes in the fluorescence intensity (d*F*/*F*) of control and ChR2‐engineered forebrain organoids. Five cells per sample, three biological replicates were evaluated in each group (*n* = 15). Significance was calculated using an unpaired *t‐*test. (f) Representative traces of calcium imaging analysis of selected cells following light stimulation of control and ChR2‐engineered forebrain organoids. (g) Quantification of stimulation‐triggered amplitudes, shown in comparison to randomized‐triggered amplitudes in control and ChR2‐engineered forebrain organoids. The median amplitude of the five pulses delivered per cell is shown. Five cells per sample, three biological replicates were evaluated in each group (*n* = 15). Significance was calculated using an unpaired *t‐*test. (h) Quantification of peak amplitudes by measuring the relative changes in the fluorescence intensity (d*F*/*F*) of day 65 control and ChR2‐engineered forebrain organoids. Five cells per sample, three biological replicates were evaluated in each group (*n* = 15). Significance was calculated using an unpaired *t‐*test. (i) Quantification of stimulation‐triggered amplitudes in control and ChR2‐engineered forebrain organoids. The median amplitude of the five pulses delivered per cell is shown. Five cells per sample, three biological replicates were evaluated in each group (*n* = 15). Significance was calculated using an unpaired *t‐*test. (j) Quantification of peak amplitudes of day 150 control and ChR2‐engineered forebrain organoids. Five cells per sample, three biological replicates were evaluated in each group (*n* = 15). Significance was calculated using an unpaired *t‐*test. (k) Quantification of the proportion of ChR2‐engineered forebrain organoids that exhibit ChR2‐expression, functional activity upon light stimulation, and viability, based on a total of 15 organoids examined on days 35, 65, and 150.

To validate the functional properties of ChR2‐engineered forebrain organoids, we performed optogenetic experiments (Figure 6a). We provided light stimulation (68 ms, 488 nm) to ChR2‐engineered forebrain organoids and examined neural activities by calcium imaging. Cal‐590 AM, a red‐shifted calcium indicator, was used to avoid optical crosstalk with the light delivered to stimulate forebrain organoids (Figure 6b). We found that light stimulation to ChR2‐engineered forebrain organoids at day 65 derived from the heterozygous line ChR2‐2 and the homozygous line ChR2‐3 both induced calcium spikes in neurons (Figure 6c). These responses were dependent on the presence of ChR2, as light stimulation to non‐targeted control organoids did not induce any responses (Figure 6c–e). To further confirm whether these neural activations are optogenetically evoked responses mediated by ChR2 upon light stimulation, we compared the stimulus‐triggered changes in amplitude (d*F*/*F*) to random time‐locked changes in the same cell. We found that the median amplitude in response to light stimulation was up to 5 times greater compared to that of the randomly selected time points in ChR2‐engineered forebrain organoids, whereas no significant difference was observed in control forebrain organoids (Figure 6d). Additionally, forebrain organoids derived from the homozygous line ChR2‐3 showed slightly higher amplitudes of calcium spikes upon light stimulation than organoids from the heterozygous line ChR2‐2 (Figure 6e).

We further examined the functional properties in response to optogenetic stimulation in long‐term cultured ChR2‐engineered forebrain organoids at day 150. Similar to our findings with day 65 organoids, we observed light‐induced calcium spikes only in ChR2‐engineered organoids at day 150, with no such responses in control organoids (Figure 6f–h). The amplitudes of these spikes were slightly elevated in organoids from the homozygous line ChR2‐3 compared to those from the heterozygous line ChR2‐2 (Figure 6f–h). Furthermore, the stimulation‐triggered median response amplitudes and peak amplitudes observed in organoids at day 150 for both the homozygous and heterozygous lines were greater than those at day 65 (Figure 6i,j), suggesting that the functional properties of the ChR2‐engineered forebrain organoids are not only maintained but potentially enhanced over an extended culture period (Figure 6k).

Altogether, these data suggest that ChR2‐engineered forebrain organoids elicit robust neural activation upon light stimulation, which provides a model system for optogenetic control of neural activities in a spatiotemporal manner to understand neural circuits and dynamics of the human brain.

### 2.6 Optogenetic stimulations of ChR2‐engineered forebrain organoids induce robust muscle contraction in forebrain–spinal cord–skeletal muscle hybrid assembloids

**FIGURE 7:**
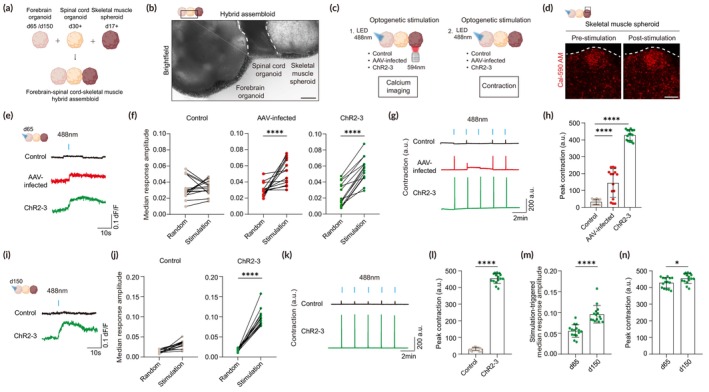
Optogenetic stimulation of ChR2‐engineered forebrain organoids to control muscle activities in forebrain‐spinal cord‐skeletal muscle hybrid assembloids. (a) Experimental scheme to generate forebrain‐spinal cord‐skeletal muscle hybrid assembloids. (b) Representative brightfield image of intact forebrain‐spinal cord‐skeletal muscle hybrid assembloids. This image was generated by tiling multiple images. Scale bar, 100 μm. (c) Schematic illustrations for optogenetic experiments. Skeletal muscle activity upon selective light stimulation on forebrain organoids within forebrain–spinal cord–skeletal muscle hybrid assembloids was examined by calcium imaging and contraction analysis. (d) Representative images of Cal‐590 AM in skeletal muscle before and after light stimulation of ChR2‐engineered forebrain organoids in forebrain–spinal cord–skeletal muscle hybrid assembloids. Scale bar, 100 μm. (e) Representative traces of calcium imaging analysis of selected cells in skeletal muscle spheroids following light stimulation of control, AAV‐infected, and ChR2‐3 hybrid assembloids with day 65 forebrain organoids. (f) Quantification of stimulation‐triggered amplitudes in skeletal muscles, shown in comparison to randomized‐triggered amplitudes. The median d*F*/*F* amplitude of the five pulses delivered per cell is shown. Five cells per sample, three biological replicates were evaluated in each group (*n* = 15). (g) Muscle contraction analyses of control, AAV‐infected, and ChR2‐3 hybrid assembloids. (h) Quantification of muscle contraction in hybrid assembloids. Spontaneous contraction was recorded for 2 min and optogenetic stimulation was given 5 times every 110 s. The highest value of displacement in the pre‐stimulation stage was measured and the average of the highest value immediately after every stimulation was quantified using MUSCLEMOTION. Five peak contractions followed by five optogenetic stimulus for three biological replicates were evaluated in each group (*n* = 15). Significance was calculated using an unpaired *t‐*test. (i) Representative traces of calcium imaging analysis of selected cells in skeletal muscle spheroids following light stimulation of control and ChR2‐3 hybrid assembloids with day 150 forebrain organoids. (j) Quantification of stimulation‐triggered amplitudes in skeletal muscles, shown in comparison to randomized‐triggered amplitudes. The median d*F*/*F* amplitude of the five pulses delivered per cell is shown. Five cells per sample, three biological replicates were evaluated in each group (*n* = 15). (k) Muscle contraction analyses of control and ChR2‐3 hybrid assembloids with day 150 forebrain organoids. (l) Quantification of muscle contraction in hybrid assembloids using MUSCLEMOTION. Five peak contractions followed by five optogenetic stimulus for three biological replicates were evaluated in each group (*n* = 15). Significance was calculated using an unpaired *t‐*test. (m) Quantification of stimulation‐triggered amplitudes in skeletal muscles in control and ChR2‐3 hybrid organoids with days 65 and 150 forebrain organoids. The median d*F*/*F* amplitude of the five pulses delivered per cell is shown. Five cells per sample, three biological replicates were evaluated in each group (*n* = 15). (n) Quantification of muscle contraction in hybrid assembloids using MUSCLEMOTION in control and ChR2‐3 hybrid assembloids with days 65 and 150 forebrain organoids. Five peak contractions followed by five optogenetic stimulus for three biological replicates were evaluated in each group (*n* = 15). Significance was calculated using an unpaired *t‐*test.

As our ChR2‐engineered forebrain organoids show homogeneous and stable expression of ChR2 throughout the entire regions of the organoids, we can drive robust and strong neural activation at once through spatial control of illumination. To test the applicability of our organoid platform to control activities of other connected tissues by optogenetically stimulating our ChR2‐engineered forebrain organoids only, we attempted to create forebrain–spinal cord–skeletal muscle hybrid assembloids that can model higher‐order control of motor activities (Figure 7a).^17^ To this end, we generated spinal cord organoids from hPSCs and characterized them using the ventral progenitor marker OLIG2 (Figure S2a–c). We confirmed the presence of OLIG2^+^ progenitors that give rise to motor neurons in spinal cord organoids (Figure S2a–c). For incorporating skeletal muscles, human skeletal myoblasts were mixed with extracellular matrix to generate 3D structures, which were then differentiated into multinucleated myotubes to form skeletal muscle spheroids (Figure S3a). Fast skeletal myosin heavy chain and laminin were highly expressed in skeletal muscles, exhibiting mature skeletal muscle identity (Figure S3b). We then fused ChR2‐engineered forebrain organoids with spinal cord organoids and skeletal muscle spheroids to generate forebrain–spinal cord–skeletal muscle hybrid assembloids (Figure 7b).

To test whether optogenetic stimulation of ChR2‐engineered forebrain organoids elicits more robust motor activity and muscle contraction within the hybrid assembloid system, we assessed skeletal muscle activity by calcium imaging and contraction analysis (Figure 7c). To compare with conventional viral delivery approach, ChR2 was separately delivered into forebrain organoids using AAV‐CAG‐ChR2‐mCherry (Figure S4a–c). These forebrain organoids were then fused with spinal cord organoids and skeletal muscle spheroids to generate hybrid assembloids. In response to optogenetic stimulation of the entire region of forebrain organoids, skeletal muscles showed increased calcium levels in both hybrid assembloids with ChR2‐3‐derived forebrain organoids (hereafter, ChR2‐3 hybrid assembloids) and AAV‐infected forebrain organoids (hereafter, AAV‐infected hybrid assembloids) but not in hybrid assembloids with control forebrain organoids without ChR2 (hereafter, control hybrid assembloids) (Figure 7d–f). In addition, the mean amplitude of calcium spikes in ChR2‐3 hybrid assembloids was higher than that of AAV‐infected hybrid assembloids (Figure 7d–f). We further examined skeletal muscle contraction upon selective stimulation of forebrain regions by quantification of muscle movement using MUSCLEMOTION.^51^ Similar to calcium imaging data, we observed global muscle contraction upon light stimulation in both ChR2‐3 and AAV‐infected hybrid assembloids but not in control hybrid assembloids (Figure 7g,h), suggesting that muscle contraction is dependent on ChR2 expression in forebrain organoids. Notably, muscle contraction responses were more consistent and robust in ChR2‐3 hybrid assembloids than in AAV‐infected hybrid assembloids (Figure 7g,h). In ChR2‐3 hybrid assembloids, all 5 light pulses all induced successful contraction responses with stronger muscle displacements, whereas AAV‐infected hybrid assembloids showed weaker and inconsistent responses upon multiple light stimuli (Figure 7g,h), which might be due to the heterogenous expression of ChR2 in AAV‐infected forebrain organoids that may reduce the number of neurons activated upon light stimulation (Figure S4b).

We further assessed the functionality of long‐term cultured ChR2‐engineered forebrain organoids within the hybrid assembloid platform. Optogenetic stimulation of 150‐day‐old forebrain organoids resulted in enhanced muscle activities and stronger muscle contractions compared to those from day 65 forebrain organoids when fused with spinal cord organoids and skeletal muscle spheroids (Figure 7i–n). These findings suggest that long‐term cultured ChR2‐engineered forebrain organoids potentially develop more mature and extensive neural projections, leading to stronger responses within the hybrid assembloid platform.

Overall, our findings demonstrated that our ChR2‐engineered forebrain organoids that homogeneously express ChR2 throughout the entire regions of the organoids can be fused with spinal cord organoids and skeletal muscle spheroids to elicit robust responses upon optogenetic stimulation, suggesting that this technology can be used to develop an in vitro platform for optogenetic experiments to better understand functional connectivity and tissue dynamics between various tissues.

## 3 MATERIALS AND METHODS

### 3.1 hPSC culture

All hPSC lines were maintained in 5% CO_2_ incubators at 37°C. hPSC lines were cultured on mitomycin C‐treated mouse embryonic fibroblasts in human hPSC medium consisting of DMEM/F12 (Gibco) supplemented with 20% KnockOut Serum Replacement (Gibco), 1× Glutamax (Gibco), 1× non‐essential amino acids (Gibco), 1% penicillin–streptomycin, 100 μM 2‐Mercaptoethanol (Sigma), and 10 ng/mL of human basic FGF (PeproTech). The cells were fed daily and passaged by manual dissection at 70% confluence. The control hPSC line IMR90 was obtained from Coriell Institute for Medical Research.

### 3.2 Cloning

For HDR, the EF1α promoter and ChR2 was cloned into a donor template. The EF1α promoter and ChR2 from pL‐CRISPR‐EFS‐GFP (Addgene #57818) and pAAV‐CAG‐hChR2‐mCherry‐WPRE‐SV40 (Addgene #100054), respectively, were amplified and cloned into pAAVS1‐P‐MCS (Addgene #80488), followed by Stbl3 transformation and further growth at 30°C in LB broth containing 100 μg/mL ampicillin. Cells were collected by centrifugation after overnight growth at 30°C.

### 3.3 Generation of ChR2‐engineered hPSC lines

The hPSCs were maintained on Matrigel (hES‐qualified, Corning)‐coated plates at 70% confluence before electroporation. One hour prior electroporation, hPSCs were treated with 10 μM Y‐27632. Electroporation was performed with the NEPA electroporator (NEPA21, CUY650P5; Poring pulse: voltage 125 V, length 2.5 ms, interval 50 ms, 2 pulses, decay rate 10%, positive polarity; Transfer pulse: voltage 20 V, length 50 ms, interval 50 ms, 5 pulses, decay rate 40%, reverse polarities). 4 × 10^5^ of hPSCs and 1.8 μg of donor template were mixed with 40 pmol of sgRNA (GGGGCCACUAGGGACAGGAUUGG) and 36 pmol of Cas9 protein (Integrated DNA Technologies (IDT)) and added to NEPA electroporation cuvettes with a 1 mm gap (NEPA). The cells were recovered immediately after electroporation with mTeSR (StemCell Technologies) containing Y‐27632, and after 48 h, the media was changed without adding the Y‐27632. Two days following transfection, 0.5 μg/mL puromycin (Sigma) was added for 7–10 days, and the medium was changed daily. After clonal expansion, the gRNA was extracted per colony using DNeasy Blood & Tissue kits (QIAGEN) and genotyped using three primers (P1 5′‐TCGACTTCCCCTCTTCCGATG‐3′, P2 5′‐CTCAGGTTCTGGGAGAGGGTAG‐3′, P3 5′‐GAGCCTAGGGCCGGGATTCTC‐3′). Sequence validation of the amplicons were done by analyzing the Sanger sequencing data.

### 3.4 Western blot

Seventy percent confluent hPSCs were collected after treatment with RIPA lysis and extraction buffer (Thermo) containing 1X Halt™ Protease Inhibitor Cocktail (Thermo). Total cell lysate (30–40 μg per lane) was separated by SDS‐PAGE (Bio‐Rad) for 2 h and transferred to 0.2 μm nitrocellulose membranes at RT for 2 h. Ponceau S staining was performed to verify the transfer of blots. Western blots were probed with anti‐ChR2 (1:500, PROGEN) and anti‐beta actin (1:5000, Santacruz), and the secondary immunoreactions were performed using horseradish peroxidase–conjugated goat anti‐mouse (1:5000, Abcam). The blots were visualized using Amersham ECL Prime (Cytiva) for 5 min until bands of interest became visible. Blots were imaged by ImageQuant 800 (Cytiva).

### 3.5 RT‐qPCR

RT‐qPCR was performed as previously described.^52^ hPSC colonies were dissociated into single cells using Accutase (Sigma), and RNA was extracted using the RNeasy Plus Mini Kit (QIAGEN). For RT‐qPCR, the cDNA was first synthesized using the first‐strand cDNA using a High‐Capacity cDNA Reverse Transcriptase Kit (Applied Biosystems) with oligo dT. Then, qPCR was performed using SYBR Green Supermix (Applied Biosystems) on a StepOnePlus Real‐Time PCR system (Applied Biosystems). Gene expression was normalized to the housekeeping gene *GAPDH*. Primers used in this study are listed in Table S1.

### 3.6 Immunofluorescence analysis

Immunofluorescence analysis was performed as previously described.^52^ Briefly, samples were fixed in 4% paraformaldehyde (PFA) for 15 min. After PBS washes, the samples were kept in 30% sucrose overnight at 4°C after fixation. Then, samples were embedded in O.C.T. compound (Leica) and frozen at −20°C. O.C.T. blocks were sectioned at 20 μm thickness using a cryostat (Leica). Frozen sections were fixed in 4% PFA for 20 min at 4°C, washed with PBS three times, and blocked in 2% goat serum and PBS containing 0.25% Triton X‐100 (PBS‐T) for 1 h at RT. Then, the sections were incubated with primary antibodies diluted in blocking buffer overnight at 4°C. The following primary antibodies were used: TUJ1 (1:300, BioLegend), ChR2 (1:100, PROGEN), Ki67 (1:500, Abcam), SOX2 (1:300, Abcam), MAP2 (1:300, Abcam), Cleaved Caspase‐3 (1:400, Cell Signaling Technology), OLIG2 (1:500, Millipore), BF‐F3 (1:500, DSHB), 6H1 (1:50, DSHB), and laminin (1:200, Sigma). Sections were washed three times with 0.25% PBS‐T and incubated with secondary antibodies (1:1000, Life Technologies) diluted in PBS‐T for 1 h. After that, sections were washed with 0.25% PBS‐T and mounted with Prolong Gold mounting reagent (Invitrogen).

For immunocytochemistry, cells were plated on Matrigel (hESC‐qualified, Corning)‐coated coverslips on a 12‐well plate. When cells reached 80% confluence, cultures were washed with PBS and fixed using 4% PFA for 5 min at RT. Cells were washed with PBS three times and blocked for 40 min. Cells were then incubated with diluted primary antibodies for 1–2 h at RT and washed three times with PBS‐T. Secondary antibodies were diluted in blocking buffer in 1:1000 (Life Technologies) for 40 min at RT. Cells were washed twice with PBS‐T and mounted on glass slides using Prolong Gold mounting reagent.

### 3.7 Calcium imaging

Calcium imaging was performed following the established protocol with some modifications.^17^ Briefly, forebrain organoids and forebrain–spinal cord–skeletal muscle hybrid assembloids were incubated with 5 μM Cal‐590 AM (AAT Bioquest) for 30 min at 37°C and washed once for 10 min with fresh medium. Then, they were transferred onto a 35‐mm confocal dish (SPL). The optogenetic stimulation was given using the ND stimulation software module of the Nikon AX confocal microscope (488 nm). Forebrain organoid‐specified ROI was drawn to stimulate the particular region only. At a frame rate of 15 frames/s, spontaneous calcium activity experiment of consistent 100 frames were acquired during pre‐stimulation and post‐stimulation with a 594 nm filter. Calcium spikes were analyzed using the ImageJ software with Time Series Analyzer V3.

### 3.8 Generation of forebrain organoids

Forebrain organoids were generated following the established protocol.^53^ In brief, colonies on mitomycin C‐treated MEF were detached after 1 h incubation with collagenase IV in 37°C. These embryoid bodies were cultured in DMEM/F12 supplemented with 20% KnockOut Serum Replacement, 1× Glutamax, 1× Non‐essential amino acids, 1% penicillin–streptomycin, 100 μM 2‐Mercaptoethanol, 10 ng/mL of human basic FGF, 2 μM dorsomorphin (Sigma), and 2 μm of A83‐01 (Tocris) for 7 days. Then, embryoid bodies were embedded in Matrigel (Growth Factor Reduced) with DMEM/F12 supplemented with 1× Glutamax, 1× Non‐essential amino acids, 1% penicillin–streptomycin, 1× N‐2 supplement (Gibco), and 10 μM SB‐431542 (Sigma) for another 7 days. At this point, rosettes were clearly visible. Next, forebrain organoids were cultured in a shaking incubator in DMEM/F12 supplemented with 1× Glutamax, 1× Non‐essential amino acids, 1% penicillin–streptomycin, 100 μM 2‐Mercaptoethanol, 1× N‐2 supplement, 1× B‐27 supplement (Gibco), and 2.5 μg/mL Insulin (Sigma). From day 35, Matrigel was mixed in the latest forebrain medium.

### 3.9 Generation of spinal cord organoids

Generation of spinal cord organoids was performed following the established protocol.^17^ Once hPSC colonies were detached using 1 mg/mL collagenase IV, hPSC medium was treated with dual SMAD inhibitors until day 5 and 3 μM of the WNT activator CHIR 99021 (Tocris) from day 4 to 18. On day 6, neural medium containing neurobasal‐A (Gibco), 1× B‐27 supplement without vitamin A (Gibco), 1× Glutamax, 1% penicillin–streptomycin and supplemented with 0.1 μM RA (Sigma), 20 ng/mL EGF (Peprotech), and 10 ng/mL human basic FGF was used with the addition of 0.1 μM SHH modulator smoothened agonist (Sigma) from day 11. From day 7, the media was changed every other day. On day 19, neural medium supplemented with 1× N‐2 supplement, 20 ng/mL BDNF (Peprotech), 10 ng/mL IGF‐1 (Peprotech), 200 nM L‐ascorbic acid (AA; Sigma), and 50 nM cAMP (Sigma) was used in culture. 2.5 μM of the Notch pathway inhibitor DAPT (Sigma) was added on days 19, 21, and 23.

### 3.10 Generation of skeletal muscle spheroids

Skeletal muscle spheroids were generated following the established protocol with some modifications.^17^ Human skeletal myoblasts were purchased from Thermo Fisher Scientific (A12555) and maintained in an undifferentiated state with Skeletal Muscle Cell Growth Medium (Promocell) in 6‐well plates (Primaria Cell Culture Dish, Corning). The medium was changed every 2–3 days, and skeletal myoblasts were passaged using 0.25% Trypsin–EDTA (Welgene). On passage 4, 3 × 10^4^ cells were resuspended with 10 μL of Matrigel (Growth Factor Reduced). By placing the drop on a clean parafilm strip, the drops were baked for 5 min at 37°C, and then cultured with the Skeletal Muscle Cell Growth Medium. After 7–10 days, the medium was changed to Skeletal Muscle Cell Differentiation Medium and was changed every other day.

### 3.11 AAV infection of forebrain organoids

For AAV infection of forebrain organoids, day 55 forebrain organoids were incubated with AAV‐CAG‐ChR2(H134R)‐mCherry‐WPRE‐SV50 (Addgene #100054‐AAV1) at the titer of 4 × 10^10^ viral genomes per mL (vg/mL) in 200 μL of media as described earlier.^20^ Here, DMEM/F12 supplemented with 1× Glutamax, 1× Non‐essential amino acids, 1% penicillin–streptomycin, 100 μM 2‐Mercaptoethanol, 1× N‐2 supplement, 1× B‐27 supplement, and 2.5 μg/mL Insulin was used in place of the neural medium. After 12 h, 800 μL of the medium was added and the forebrain organoids were washed with fresh medium the next day.

### 3.12 Generation of forebrain–spinal cord–skeletal muscle hybrid assembloids

Forebrain–spinal cord–skeletal muscle hybrid assembloids were generated following the established protocol.^17^ Briefly, skeletal muscle spheroids were cultured in differentiation medium (Promocell) for at least 10 days and spinal cord organoids over 25 days were closely placed on transwells (0.4 μm pore size, SPL) in 12‐well plates. Half of the DMEM/F12 medium supplemented with 1% non‐essential amino acids, 1% Insulin‐Transferrin‐Selenium (Thermo), 1% penicillin–streptomycin, 200 nM L‐ascorbic acid, and 50 nM cAMP were changed every other day. One to 2 days later, the forebrain organoids were placed next to the spinal cord organoids. Control, AAV‐infected, and ChR2‐engineered forebrain organoids on days 62–65 or day 150 were fused with spinal cord‐skeletal muscle hybrid assembloids. Days 25–30 spinal cord organoids and skeletal muscle spheroids cultured for more than 17 days were used in the experiments.

### 3.13 Contraction analysis

Using the automated, open‐source ImageJ plugin MUSCLEMOTION^51^ (https://github.com/l-sala/MUSCLEMOTION), contractions of human skeletal muscle were quantified. MUSCLEMOTION quantifies motion by subtracting the summed pixel intensity changes between a reference frame and the frame of interest. For the quantification of hybrid assembloids stimulation experiments, the pixel intensity analysis was performed as described above using MUSCLEMOTION.

### 3.14 Statistical analysis

All data were expressed as mean ± standard error of the mean (SEM). The results were analyzed using GraphPad Prism ver. 9. Comparisons between groups were performed using an unpaired *t‐*test or a nested *t‐*test as described in figure legends. Differences were considered significant when the values of *p* were less than .05.

## 4 CONCLUSIONS

A major conceptual advance presented in our current study is the development of genetically engineered human forebrain organoids that homogeneously express ChR2 during long‐term culture, allowing for the non‐virus mediated, spatiotemporal optogenetic control of neural activities in human forebrain organoids. To achieve this, the *ChR2* gene was targeted into the *AAVS1* safe harbor locus of hPSCs, which were further differentiated into mature human forebrain organoids. Through this innovative technology, we were able to create forebrain organoids that stably express ChR2 throughout entire regions of the organoids without any structural and functional perturbation. These newly conceived forebrain organoids showed a non‐virus mediated, robust neural activation upon light stimulation and further induced strong and consistent muscle contraction in the hybrid assembloid platform, being connected to spinal cord organoids and skeletal muscles spheroids. To our knowledge, such efforts to generate genetically engineered forebrain organoids with homogeneous, stable, and long‐term expression of ChR2 have not been previously described. Generation of such organoids is particularly relevant to modern research because the importance of developing human brain model systems with technically easier spatiotemporal optogenetic control of neural activities to understand human neural networks and their dynamics is increasingly being recognized. Therefore, our ChR2‐expressing, genetically engineered forebrain organoids will serve as an innovative experimental platform to study neural circuits in normal as well as patient‐specific human brains with various neurological disorders.

It is important to note that one of the conceptual advances in our study is the targeted introduction of the *ChR2* gene into the *AAVS1* locus for optogenetic control. The *AAVS1* locus, which is located in human chromosome 19 and is part of the *PPP1R12C*, was originally described as a main hotspot for AAV integration.^44^, ^45^, ^46^, ^47^ Targeted integration of transgenes into the *AAVS1* locus not only allows for stable expression of introduced transgenes in various cell types, including hPSCs,^44^, ^45^, ^46^, ^47^ but is also considered a safe harbor site, as disruption of *PPP1R12C* is not linked to any known disease.^44^, ^45^ In our study, targeted introduction of the *ChR2* gene into the *AAVS1* locus allowed homogeneous and stable expression of ChR2 in hPSCs, whose expression was further maintained throughout the differentiation process. Using ChR2‐engineered hPSCs, we were able to create forebrain organoids with homogeneous expression of ChR2 throughout entire neurons, which provides ease of spatiotemporal control of neural activities in response to light stimulation. Observing neural activities in several parts of the brain organoids while giving optogenetic stimulation within restricted regions helps to understand neural networks and dynamics in the human brain as well as in the context of various neurological diseases such as schizophrenia, autism, and Alzheimer's disease.^27^, ^54^, ^55^, ^56^, ^57^

Another important point of our study is that ChR2‐engineered forebrain organoids may serve as robust systems to model neural connectivity and the dynamics between various types of tissues. Cortico‐spinal‐motor hybrid assembloids,^17^ in which ChR2‐engineered forebrain organoids are fused with spinal cord organoids and skeletal muscle spheroids, showed stronger and more consistent muscle contraction upon light stimulation of forebrain organoids than hybrid assembloids consisting of forebrain organoids with ChR2 introduced via viral delivery. The homogeneous and stable expression of ChR2 throughout the entire neuron that functionally connect to spinal cord organoids contributes to the robust contraction of skeletal muscles. By controlling the neural activity in ChR2‐engineered forebrain organoids using light and observing the activity of other tissues within hybrid assembloid systems, where ChR2‐engineered forebrain organoids are fused with various distinct tissues representing other brain regions or peripheral organs, our ChR2‐engineered forebrain organoids will provide a powerful tool to regulate the activity of other connected tissues and deepen the understanding of neural connectivity and tissue dynamics between different brain regions and various tissues. Moreover, using patient‐derived iPSCs, our study may further facilitate the establishment of an innovative model platform to study a range of human diseases at molecular and cellular levels where neural networks and tissue connections play key roles in the pathogenesis.^26^, ^27^, ^55^, ^56^, ^57^, ^58^, ^59^, ^60^

In this study, our ChR2‐engineering platform was validated within a long‐term culture system. The 150‐day‐old, ChR2‐engineered forebrain organoids not only displayed mature neuronal morphologies but also maintained stable and consistent ChR2 expression. Moreover, these forebrain organoids exhibited enhanced functionality upon light stimulation and elicited robust and strong muscle contraction within a hybrid platform when fused with spinal cord organoids and skeletal muscle spheroids. Despite some necrotic regions developing in the core as the organoids increased in size during the extended culture period, their overall viability and functionality were not compromised. Thus, using mature, long‐term cultured forebrain organoids might be valuable for optogenetic control of neural activities in further experiments.

Moving forward, there are several potential applications for technical advances in this system. Recent studies have demonstrated that forebrain organoids cultured in a scaffold matrix, such as human brain tissue‐derived brain extracellular matrix (ECM), exhibited enhanced neuronal differentiation, improved cortical organization, and functional maturation.^61^, ^62^ Additionally, the integration of a microfluidic device with brain‐specific ECM further increased neuronal cell expansion and reduced cellular apoptosis by improving oxygen supply and nutrient exchange.^61^, ^62^ Applying these tissue‐engineering platforms, including brain‐specific ECM and microfluidic devices, to our ChR2‐engineering system may enhance the growth and maturation of ChR2‐engineered forebrain organoids as well as hybrid assembloids, which will allow more robust and effective optogenetic control of the ChR2‐engineered forebrain organoids.

Although ChR2‐engineered forebrain organoids developed in our study showed robust neural activation upon light stimulation, neural activities cannot be suppressed or controlled in certain populations. The selective control of neural activities can be achieved by integrating control elements such as promoters, enhancers, and targeting sequences together with ChR2, allowing specific expression of ChR2 in certain populations.^28^, ^29^, ^34^ Combination with other types of light‐sensitive proteins with different functions and sensitivity to certain wavelengths will further allow for both activation and inhibition of neural activities in specific populations within one system using different wavelengths of light.^17^, ^30^, ^35^

Taken together, our study, which has the potential to profoundly affect our understanding of neural circuits and dynamics between different regions of the brain and various tissues, may provide a unique experimental tool for studying neurobiology in the human brain and a range of neurological diseases at molecular and cellular levels, for which understanding of pathogenesis requires an organoid system where neural activities can be robustly controlled in a spatiotemporal manner. This system can be further utilized in other fields such as cardiology to understand cardiomyocyte contraction and to develop new therapeutic options to synchronize cardiac pacing.^63^ Overall, our study will facilitate a broader spectrum of basic studies and translational research, from exploring neural circuits in the human brain to establishing novel personalized therapeutic options, such as new drug screening strategies that are customized for patients with various diseases with defective neural connections and dynamics.

## AUTHOR CONTRIBUTIONS

**Soojung Hong:** Conceptualization; data curation; formal analysis; investigation; methodology; project administration; resources; software; validation; visualization; writing – original draft. **Juhee Lee:** Methodology. **Yunhee Kim:** Methodology. **Eunjee Kim:** Funding acquisition; project administration; supervision; writing – original draft; writing – review and editing. **Kunyoo Shin:** Funding acquisition; project administration; supervision; writing – original draft; writing – review and editing.

## CONFLICT OF INTEREST STATEMENT

The authors have no potential conflicts of interest to disclose.

## Supporting information


**FIGURE S1.** Apoptosis analysis of ChR2‐engineered forebrain organoids during long‐term culture. (a–c) Representative images of forebrain organoids at day 35 (a), day 65 (b), and day 150 (c) immunostained for cleaved caspase‐3. Scale bars, 100 μm. (d) Quantification of cleaved caspase‐3^+^ cells out of the total cells on control and ChR2‐engineered forebrain organoids on days 35, 65 and 150. Five sections per sample, three biological replicates in each group were evaluated (*n* = 15). Significance was calculated using an unpaired *t‐*test.
**FIGURE S2.** Generation of spinal cord organoids. (a) Experimental scheme to generate spinal cord organoids. Representative brightfield images of the hPSC colony and spinal cord organoids at days 1, 5, 8, 18, and 25 are shown below. Scale bar, 100 μm. (b) Representative images of spinal cord organoids at day 18 immunostained for OLIG2. Scale bar, 100 μm for upper panels and 50 μm for lower panels. (c) Quantification of OLIG2^+^ cells in spinal cord organoids. Two sections per sample, three biological replicates were evaluated in each group (*n* = 6). Significance was calculated using an unpaired *t‐*test.
**FIGURE S3.** Generation of skeletal muscle spheroids. (a) Schematic illustration to generate skeletal muscle spheroids from human skeletal myoblasts. Representative brightfield images of skeletal myofibroblasts and skeletal muscle spheroids at days 1, 7, and 14 are shown below. Scale bar, 100 μm. (b) Representative images of skeletal muscle spheroids at day 30 immunostained for fast MyHC (BF‐F3, 6H1) and laminin. Scale bar, 50 μm.
**FIGURE S4.** ChR2 delivery into forebrain organoids using AAV. (a) Schematic illustration to deliver ChR2 into forebrain organoids using AAV. (b) Representative images of mCherry in AAV‐infected forebrain organoids compared to non‐infected organoids after 7 days of infection. Scale bar, 100 μm. (c) Quantification of mCherry^+^ cells in AAV‐infected forebrain organoids. Three ROIs per sample, three biological replicates were evaluated in each group (*n* = 9). Significance was calculated using an unpaired *t‐*test.

**TABLE S1.** Primer sequences for RT‐qPCR and PCR genotyping.

## Data Availability

The datasets used and/or analyzed during the current study are available from the corresponding authors upon request.
