# Supplementary figures and images for: AAVS1 ‐targeted, stable expression of ChR2 in human brain organoids for consistent optogenetic control
Source: Bioeng Transl Med. 2024 Jun 9;9(6):e10690. doi: 10.1002/btm2.10690 (PMC11558186; doi:10.1002/btm2.10690)

**A**

Control

Chr2-2

Chr2-3

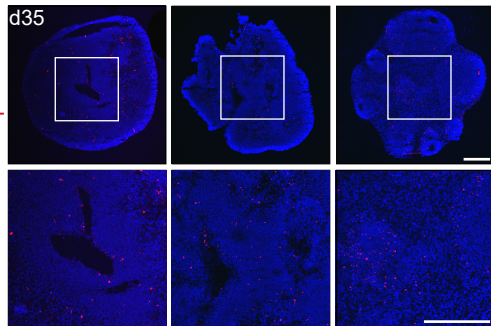**B**

Control

Chr2-2

Chr2-3

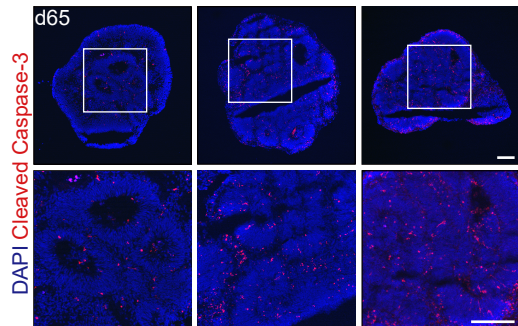**C**

Control

Chr2-2

Chr2-3

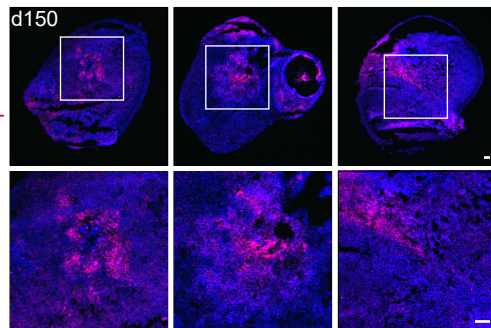**D**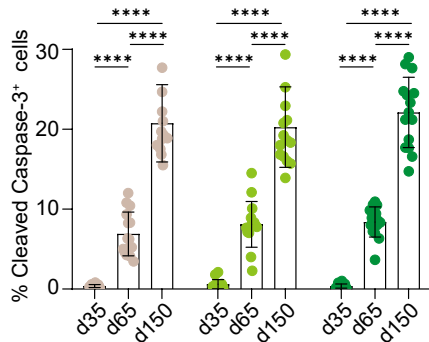

**A**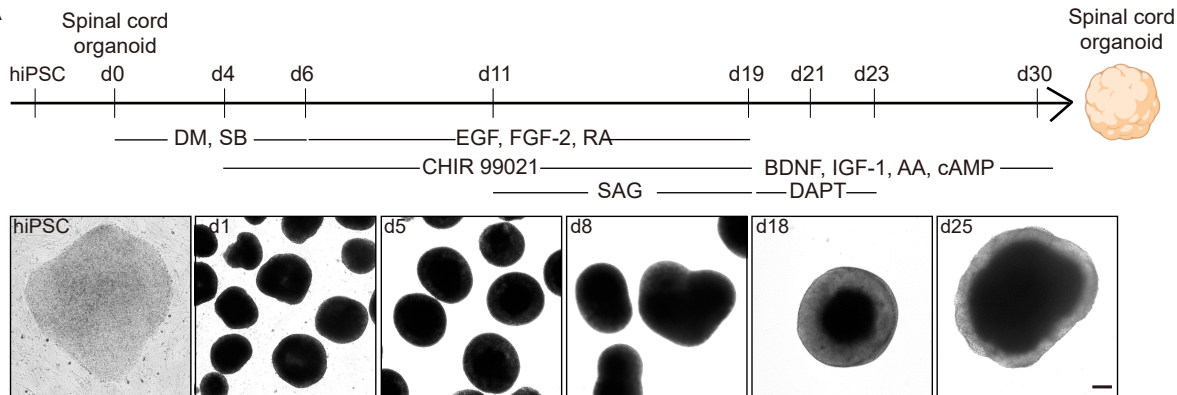**B**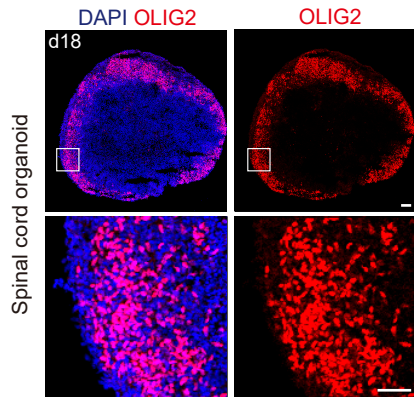**C**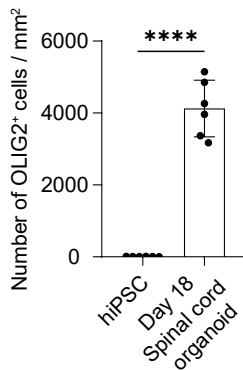

**A**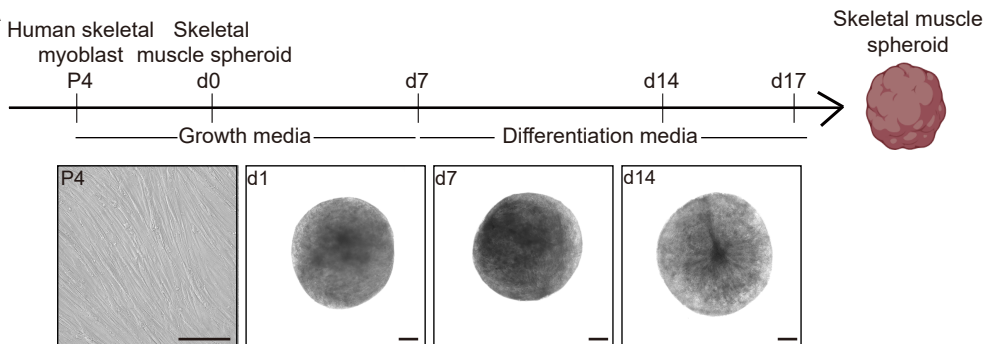**B**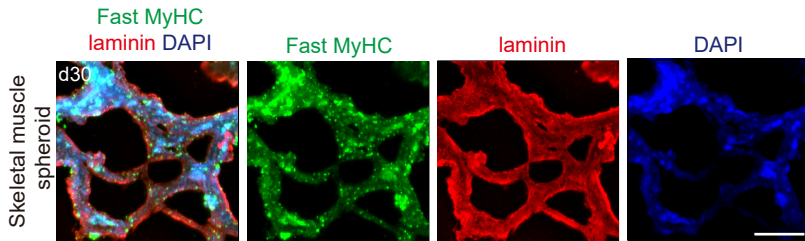

**A**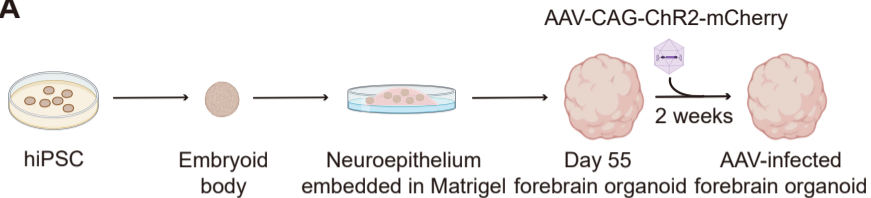**B**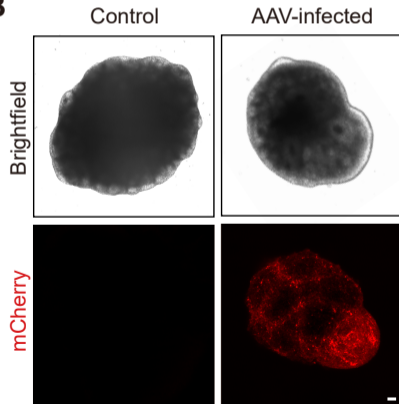**C**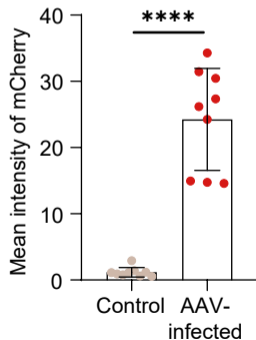

Supplement: Supplementary file 1 — FIGURE S1. Apoptosis analysis of ChR2‐engineered forebrain organoids during long‐term culture. (a–c) Representative images of forebrain organoids at day 35 (a), day 65 (b), and day 150 (c) immunostained for cleaved caspase‐3. Scale bars, 100 μm. (d) Quantification of cleaved caspase‐3+ cells out of the total cells on control and ChR2‐engineered forebrain organoids on days 35, 65 and 150. Five sections per sample, three biological replicates in each group were evaluated (n = 15). Significance was calculated using an unpaired t‐test. FIGURE S2. Generation of spinal cord organoids. (a) Experimental scheme to generate spinal cord organoids. Representative brightfield images of the hPSC colony and spinal cord organoids at days 1, 5, 8, 18, and 25 are shown below. Scale bar, 100 μm. (b) Representative images of spinal cord organoids at day 18 immunostained for OLIG2. Scale bar, 100 μm for upper panels and 50 μm for lower panels. (c) Quantification of OLIG2+ cells in spinal cord organoids. Two sections per sample, three biological replicates were evaluated in each group (n = 6). Significance was calculated using an unpaired t‐test. FIGURE S3. Generation of skeletal muscle spheroids. (a) Schematic illustration to generate skeletal muscle spheroids from human skeletal myoblasts. Representative brightfield images of skeletal myofibroblasts and skeletal muscle spheroids at days 1, 7, and 14 are shown below. Scale bar, 100 μm. (b) Representative images of skeletal muscle spheroids at day 30 immunostained for fast MyHC (BF‐F3, 6H1) and laminin. Scale bar, 50 μm. FIGURE S4. ChR2 delivery into forebrain organoids using AAV. (a) Schematic illustration to deliver ChR2 into forebrain organoids using AAV. (b) Representative images of mCherry in AAV‐infected forebrain organoids compared to non‐infected organoids after 7 days of infection. Scale bar, 100 μm. (c) Quantification of mCherry+ cells in AAV‐infected forebrain organoids. Three ROIs per sample, three biological replicates were [file BTM2-9-e10690-s001.pdf]
