# Supplementary material for: AAVS1 ‐targeted, stable expression of ChR2 in human brain organoids for consistent optogenetic control
Source: Bioeng Transl Med. 2024 Jun 9;9(6):e10690. doi: 10.1002/btm2.10690 (PMC11558186; doi:10.1002/btm2.10690)
Supplement: Supplementary file 2 — TABLE S1. Primer sequences for RT‐qPCR and PCR genotyping. [file BTM2-9-e10690-s002.pdf]

	Gene/Primer name	Primer
PCR	P1	TCGACTTCCCCTCTTCCGATG
	P2	CTCAGGTTCTGGGAGAGGGTAG
	P3	GAGCCTAGGGCCGGGATTCTC
qRT-PCR	GAPDH	Forward: GTCAAGGCTGAGAACGGGAA
		Reverse: TCGCCCCACTTGATTTTGGA
	ChR2	Forward: CAATGTTACTGTGCCGGATG
		Reverse: ATTTCAATCGCGCACACATA
